# Transcranial direct current stimulation eliminates the own-age bias as indexed by the face inversion effect for own- versus other-age faces

**DOI:** 10.1093/scan/nsag001

**Published:** 2026-01-09

**Authors:** Ciro Civile, Guangtong Wang

**Affiliations:** Department of Psychology, Faculty of Health & Life Sciences, University of Exeter, Exeter, EX4 4QG, United Kingdom; Department of Psychology, Faculty of Health & Life Sciences, University of Exeter, Exeter, EX4 4QG, United Kingdom

**Keywords:** own age bias, face inversion effect, transcranial direct current stimulation, face recognition, perceptual learning

## Abstract

We investigate individuals’ reduced ability to recognize faces of other ages, a phenomenon known as the Own-Age Bias (OAB). This study utilized a double-blind, between-subjects design in which anodal tDCS (10 minutes duration, 1.5 mA intensity, targeting the Fp3 location) was applied to disrupt the face inversion effect (FIE), which reflects higher recognition performance for upright compared to upside-down faces. Young adults participated in an old/new recognition task, where upright and inverted ‘own-age’ faces (19–30 years) and ‘other-age’ faces (older, 69–80 years) were presented. In the sham/control condition (n = 24), we observed a robust OAB, indicated by a significantly larger FIE for own-age faces compared to other-age faces. Importantly, in the anodal tDCS condition (n = 24), the FIE for own-age faces was significantly reduced compared to the sham condition, effectively eliminating the cross-age interaction index of the OAB. We interpret our results through a perceptual expertise-based account of the OAB.

## Introduction

Numerous studies in the face recognition literature have demonstrated that individuals exhibit enhanced recognition of faces within their own-age group compared to faces from other-age groups, a robust phenomenon known as the Own-Age Bias (OAB) (Anastasi and Rhodes 2006, Hills and Lewis 2011, Rhodes and Anastasi 2012, Wiese et al. 2013, Cronin et al. 2021, Strickland-Hughes et al. 2020). A central debate regarding the OAB concerns its underlying nature, whether it arises from social motivations or perceptual expertise, paralleling broader discussions on ‘own’ biases in face recognition. The most extensively studied phenomenon in this context is the Own-Race Bias (ORB) (Slone et al. 2000, Meissner and Brigham 2001, McKone et al. 2019).

Over time, various motivational accounts linked to social categorization have been proposed to explain the ORB. The most comprehensive recent explanation suggests that the ORB reflects a tendency for individuals to think categorically about outgroup members, leading to different processing of facial features compared to ingroup members. Specifically, ingroup faces (e.g. own-race) prompt perceivers to focus on distinguishing features within their group, whereas outgroup faces (e.g. other-race) are often categorized based on prototypical features, such as race, age or age, that are common across all outgroup faces. This categorization complicates individual differentiation. Therefore, it is argued that social categorizations influence how facial features are used for recognition, and this process similarly applies to categories like sex and age, thus contributing to the development of the OAB (Levin 2000, Hugenberg et al. 2013, Young et al. 2012).

A recent example of this is the work conducted by Rollins et al. (2020), in which young adults participated in an old/new recognition task. They were asked to memorize a set of faces belonging either to their own age group or to another age group (children). Subsequently, they performed a recognition task that presented the previously seen faces intermixed with new ones. The critical manipulation involved the instructions given during the learning phase. When participants were instructed to make verbal judgements about the age of faces, the recognition results revealed a robust OAB, with better recognition for own- versus other-age faces. In contrast, when participants were instructed to make gender judgements about the faces, the OAB was reduced, though still present. Although these findings are interesting, they do not definitively support a motivational account. The reduced OAB observed could simply be due to the gender-judgement instructions acting as a distraction, making face memorization more difficult, as indicated by the decreased performance for own-age faces.

Alternatively, cognitive scientists have proposed a perceptual expertise account, suggesting that the ‘own’ bias results from limited visual experience with faces of other races or ages, leading to diminished perceptual expertise (Zhao et al. 2014).

Research involving children’s OAB found that 3-year-olds recognize young adult faces more accurately than those of newborns and peers. Although not directly tested, this lack of OAB was interpreted due to more frequent interactions with adult faces, which aids in forming attachments to primary caregivers, usually young adults, supporting the perceptual expertise explanation (Cassia et al. 2009, Cassia et al. 2012). Subsequent studies suggest the OAB decreases in people with frequent contact with other age groups, like those working with children or infants (Harrison and Hole 2009), or individuals exposed to various age groups in their daily lives (Wiese et al. 2012, 2013). However, the precise mechanism by which contact improves facial recognition remains unclear, and this perceptual expertise contact-based hypothesis does not exclude the possibility that social categorization could explain the observed results.

A widely used paradigm to test the perceptual expertise hypothesis in face recognition research is the Face Inversion Effect (FIE), which refers to a decline in recognition performance when faces are presented upside down (Yin 1969, Valentine 1988, Farah et al. 1995; Busigny and Rossion 2010). Research suggests that inversion disrupts our expertise to process configural information, the spatial relationships among the facial features, resulting in reduced recognition as demonstrated by the inversion effect observed with novel artificial stimuli, such as Greebles or checkerboards, that participants become familiar with during experiments (e.g. Gauthier and Tarr 1997; McLaren 1997; Civile et al. 2014b, 2024a). Importantly, several studies have shown that manipulating facial configural information, through, manipulations such as Thatcherization or scrambling, leads to a reduction in the FIE, particularly affecting recognition of upright faces (Maurer et al. 2002; Latinus and Taylor 2005; Civile et al. 2014a, 2016a; Schwiedrzik et al. 2018; McCourt et al. 2023).

A few studies have used the FIE to investigate perceptual expertise in the OAB. For example, Kuefner et al. (2008) demonstrated that young adults exhibited enhanced recognition of upright own-age faces compared to both newborn and child faces; however, this own-age advantage disappeared when faces were presented inverted. In another study, Macchi et al. (2009) found a similar FIE for newborn and adult faces among nurses working in a maternity ward, individuals with substantial expertise in recognizing newborn faces, while novices did not show an FIE for newborn faces.

In a recent study, Civile and Wang (2025) demonstrated a robust OAB among young adults (18–30 years old), with the FIE significantly reduced for older faces due to decreased recognition of upright older faces (for similar results, see also Wang and Civile 2025). Critically, older adults (69–80 years old) did not display an OAB; the FIE for older faces was significantly larger than that found in younger adults, while the FIE for younger faces remained robust across both groups. Civile and Wang’s (2025) findings suggest that, unlike ORB, which pertains to individuals whose ingroup membership remains constant, the OAB is unique because ageing causes individuals to transition from one age group (e.g. young adults) to another (e.g. older adults). This shift implies that individuals who have developed expertise in recognizing faces of different ages, due to prior membership in those groups, are less likely to exhibit the OAB.

Collectively, these studies using the FIE to measure the OAB suggest that the FIE is diminished for other-age faces when sufficient expertise in processing configural information for upright other-age faces has not been developed. Conversely, since inversion expertise is not applicable (as we are not familiar with upside-down faces), no differences are observed between inverted own-age and other-age faces. However, these results could also be explained by social accounts. The tendency to categorize outgroup members based on prototypical elements (e.g. age) primarily affects the recognition of upright faces, leading to a reduced FIE for other-age faces. Nonetheless, from a purely social account perspective, it remains unclear why only upright faces, and not inverted other-age faces, would be affected. Importantly, Civile and Wang’s (2025) findings are challenging to explain solely from a social perspective, as they demonstrate how established perceptual expertise can eliminate the OAB by enhancing the FIE for other-age faces.

In the present study, we aimed to investigate further the nature of the OAB, this time by directly suppressing the perceptual expertise component for own-age faces and examine whether this eliminates the OAB. To achieve this, we employed a tDCS procedure, recently developed in literature, designed to disrupt the perceptual learning component of the FIE. Through a double-blind, between-subjects design, Civile et al. (2016b) provided the first evidence that anodal tDCS delivered over the left dorsolateral prefrontal cortex (DLPFC) at the Fp3 area can modulate the robust inversion effect previously established with checkerboards, a hallmark of perceptual learning (McLaren and Civile 2011; Civile et al. 2014b). Specifically, the checkerboard inversion effect in the anodal group was significantly reduced compared to the strong inversion effect observed in the sham group. The targeted brain region was selected based on prior fMRI studies showing high activation during learning tasks involving checkerboards and other configuration-based objects (Seger et al. 2000; Garcin et al. 2012), as well as previous tDCS studies that used the same procedure to modulate categorization learning of patterns (Ambrus et al. 2011) and checkerboard stimuli, though without testing an inversion effect (McLaren et al. 2016).

Further studies demonstrated that applying the same tDCS protocol to the inversion effect for faces produces similar results, showing a reduced FIE in the anodal group compared to the sham group (Civile et al. 2018). This effect has been replicated and extended in multiple publications (Civile et al. 2019, 2020a, 2020b, 2021a, 2021b, 2024b) and validated through active control studies where the same behavioural paradigm was used, but with stimulation targeting different scalp areas (Civile et al. 2021b, 2024a). This work helped establish a causal link between the inversion effects for faces and checkerboards, demonstrating that tDCS can systematically reduce both phenomena.

More recently, Civile and McLaren (2022) showed that applying the same tDCS procedure eliminated the ORB. Western Caucasian participants were assigned to either an anodal or sham/control condition before completing an old/new recognition task involving both upright and inverted Western Caucasian and East Asian faces. The sham group confirmed a robust ORB, characterized by a smaller FIE for other-race faces, due to poorer recognition of upright other-race faces. Importantly, under the anodal tDCS condition, the interaction indicating the ORB was abolished, as the FIE for own-race faces was reduced to the level observed for other-race faces. This provided evidence that disrupting perceptual expertise can effectively mitigate the ORB, representing a significant advancement in understanding the mechanisms underlying this bias.

One could argue that the effects of tDCS on the ORB may be explained by a social account. However, this would require an established link between the specific tDCS procedure and social face categorization, which has not yet been developed in the literature. Additionally, it is important to consider the causal link demonstrated in the literature between the checkerboard inversion effect, which does not involve any social aspects, and the FIE. Therefore, a social account attempting to explain the effects found by Civile and McLaren (2022) on the ORB would also need to address how these effects relate to the checkerboard inversion effect, an index of expertise development.

In our current study, we extend the use of the tDCS procedure to the OAB. Whereas in Civile and Wang (2025) the OAB was eliminated by an enhanced FIE for other-age faces, led by higher recognition of upright faces, in our study, in line with Civile and McLaren (2022), we aim to eliminate the OAB by means of reduced FIE for the ‘own’ faces, in this case own-age faces, led by a lower recognition for upright faces.

## Method 

### Participants

Overall, 48 naïve, self-declared Western Caucasian participants (36 women; mean age = 20.9 years, age range = 18–27 years) took part in the study. We only recruited Western Caucasian participants and used face stimuli of Western Caucasian targets to control for the influence of the ORB. All participants were students at the University of Exeter and were selected according to the safety screening criteria for tDCS.

The sample size was determined through an a priori power analysis conducted using G*Power software (Faul et al. 2007). The analysis was based on a 2 × 2 × 2 design (Orientation [upright, inverted] × Face Age [younger, older] × tDCS [anodal, sham]), targeting a medium effect size of f = 0.25 consistent with previous studies that employed same tDCS protocols and FIE behavioural task (e.g. Civile et al. 2018). The goal was to achieve a power greater than 0.80, which suggested a total sample size of 48.

### Materials

The study employed the set of stimuli from Civile and Wang (2025), which demonstrated an OAB indexed by the FIE. This set consists of high-resolution Western Caucasian faces from the FACES ­database (https://faces.mpdl.mpg.de/imeji; Ebner et al. 2010) and includes neutral-expression images of younger (aged 19–30 years) and older individuals (aged 69–80 years) (see the Supplemental Material Document for further details about the FACES). Fifty-six images per group, with an equal number of male and female faces, were used, and inverted faces were created by rotating the original images by 180 degrees. The images were displayed centrally on the screen (following a fixation cue) at a size of 5.5 x 7 cm. The experiment was conducted using the Gorilla experiment platform on an iMac computer, with participants seated approximately 70 cm away from the screen.

### Behavioural task

The study employed an old/new recognition as that used by Civile and Wang (2025). During the study phase, each participant was shown with 28 upright faces (14 younger and 14 older) and 28 inverted faces (14 younger and 14 older). Faces were presented one at a time in a randomized order, with no response required from the participant. Participants were instructed that a series of faces would appear one at a time and were encouraged to try to memorize them. Each trial began with a fixation cross displayed at the centre of the screen for 1 second, followed by the presentation of a face stimulus for 3 seconds. After all 56 faces had been shown, the program displayed instructions for the recognition phase.

In the recognition phase, 56 novel faces (half upright and half inverted) were added to the 56 faces previously seen. All 112 faces were presented sequentially in a randomized order, and participants were asked to press the ‘.’ key if they recognized the face as having been shown earlier, or the ‘x’ key if they did not. Key assignments were counterbalanced across participant groups. Each trial started with a fixation cross at the centre of the screen for 1 second, followed by the face stimulus for a maximum of 4 seconds within which responses had to be made. If no response was recorded within this time, the trial timed out, a ‘Too Slow’ feedback message appeared, and the next trial began automatically.

During the entire experiment, each face stimulus appeared in only one orientation per participant. Across different participant groups, each stimulus was presented in every condition, upright seen, inverted seen, upright not seen, and inverted not seen.

### tDCS apparatus

The stimulation was delivered using a battery-powered, constant-current stimulator by Neuroelectrics (https://www.neuroelectrics.com), via a pair of surface sponge electrodes (35 cm^2^), soaked in saline solution and placed on the scalp at the target stimulation sites. We adopted a bilateral bipolar non-balanced montage, with one electrode (the anode) positioned over the target area (Fp3) and the other (the return) placed on the contralateral supraorbital area, above the right eyebrow (Fp2) (Civile et al. 2021a, Civile and McLaren 2022). The procedure was conducted relying on the system’s double-blind functionality.

In the active (anodal) condition, a direct current of 1.5 mA was applied for 10 minutes, with a 5-second fade-in and a 5-second fade-out, starting as soon as participants began the learning/study phase, which lasted approximately 4 minutes.

The stimulation continued into the recognition phase, which lasted approximately 10 minutes. It is important to note that the tDCS effects persist for up to 120 minutes post-stimulation (Nitsche and Paulus 2000, Jamil et al. 2017). Additionally, no differences in the FIE have been found between stimulation delivered during the study phase and that delivered only during the recognition phase (Civile et al. 2020c).

In the sham condition, participants experienced the same initial 5-second fade-in and 5-second fade-out, but no stimulation was delivered in between.

## Results

### Data analysis

Our primary measure was performance accuracy in the old/new recognition task, which was used to compute a d-prime (d’) sensitivity measure for each condition, extracted according to the procedure established by Stanislaw and Todorov (1999). We assessed performance for each stimulus condition within each tDCS sample against the chance level (all *P* < .001) and analysed the data for criterion, C, which, in accordance with previous studies, revealed no effects of tDCS (Civile and McLaren 2022).

For completeness, details on d’ calculations, as well as the full results for the chance performance analysis, C analysis are fully reported in the Supplemental Material Document.

For the main d’ analysis we conducted a 2 × 2 × 2 mixed-model ANOVA with Orientation (upright, inverted) and Face Age (younger, older) as within-subjects factors, and tDCS Stimulation (sham, anodal) as a between-subjects factor. This revealed a significant main effect of Orientation, F(1, 46) = 40.8, *P* < .001, η^2^ₚ = .47, indicating the FIE. There was a significant main effect of Face Age, F(1, 46) = 6.65, *P* = .013, η^2^ₚ = .13, with younger faces (M = 1.10, SD = .37) recognized better overall than older faces (M = .90, SD = .34). Consistent with previous research, no significant main effect of tDCS Stimulation was found, F(1, 46) = .09, *P* = .76, η^2^ₚ < .01, confirming that tDCS does not simply influence overall performance.

Additionally, no significant interactions were observed for ­Orientation × tDCS Stimulation, F(1, 46) = 1.78, *P* = .18, η^2^ₚ = .03; Orientation × Face Age, F(1, 46) = 1.83, *P* = .18, η^2^ₚ = .04; or Face Age × tDCS, F(1, 46) = 0.10, *P* = .99, η^2^ₚ < .01. Critically, the three-way interaction—Orientation × Face Age × tDCS—was significant, F(1, 46) = 9.14, *P* = .004, η^2^ₚ = .17. To interpret this interaction, we examined the two-way interactions (Orientation × Face Age) separately within each tDCS condition.

#### Sham tDCS group

**Figure 1.**
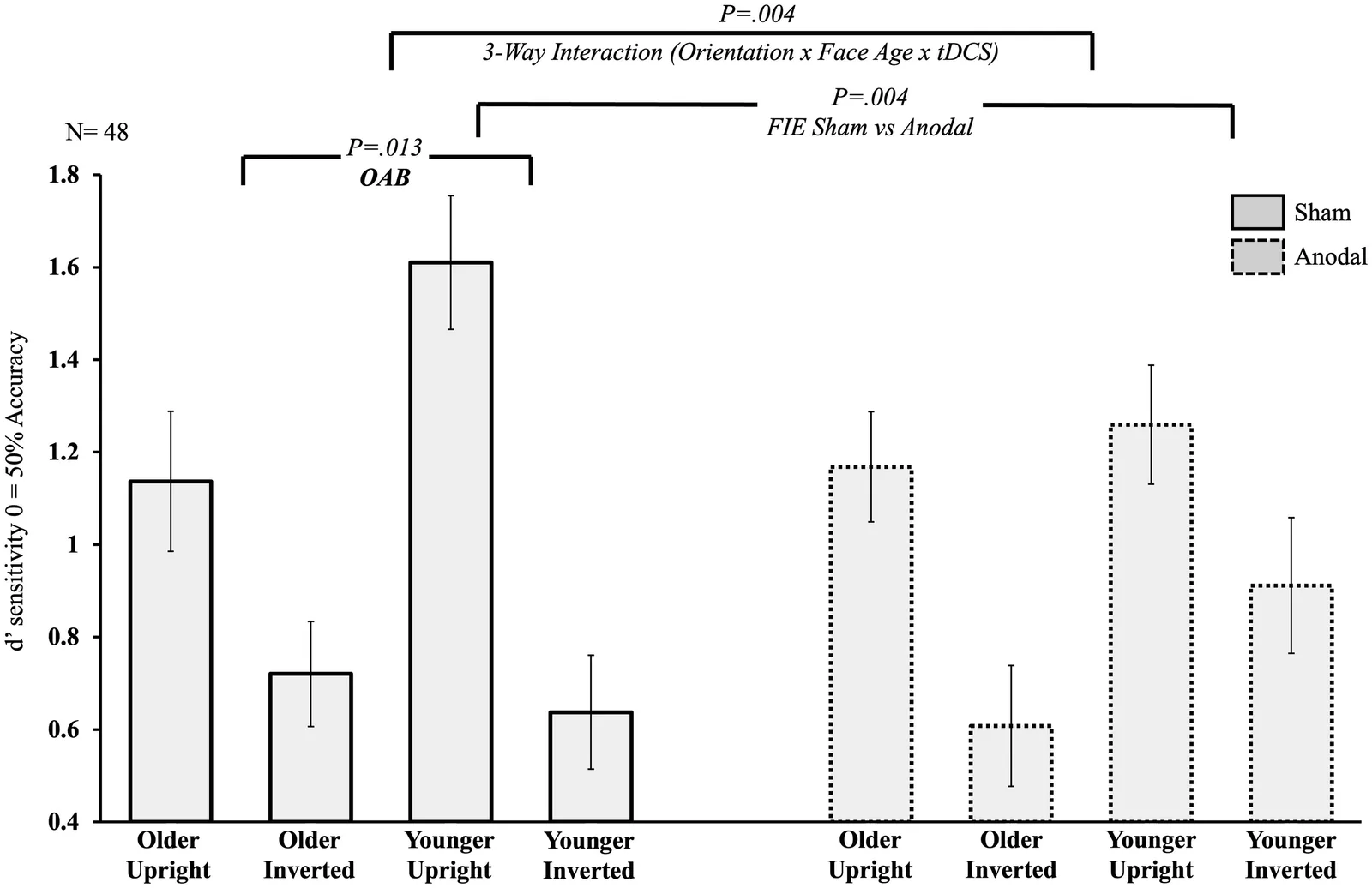
Reports the results from the study. The *x*-axis shows the face types in each tDCS group. The *y*-axis shows sensitivity *d’* measure. Error bars represent s.e.m.

A 2 × 2 ANOVA revealed a significant main effect of Orientation, F(1, 23) = 21.13, *P* < .001, η^2^ₚ = .48, indicating the FIE. There was no significant main effect of Face Age, F(1, 23) = 3.44, *P* = .076, η^2^ₚ = .13. Importantly, a significant interaction was observed, F(1, 23) = 7.19, *P* = .013, η^2^ₚ = .24, with a larger FIE for younger faces (M = .97, SD = .81) compared to that for older faces (M = .41, SD = .97), confirming the OAB. Recognition performance for upright younger faces was significantly better than for upright older faces, t(23) = 3.09, *P* = .005, η^2^ₚ = .29. No difference emerged between inverted younger and older faces, t(23) = .57, *P* = .56, η^2^ₚ = .01 (see Fig. 1).

#### Anodal tDCS group

A 2 × 2 ANOVA revealed a significant main effect of Orientation, F(1, 23) = 21.83, *P* < .001, η^2^ₚ = .48, but no significant main effect of Face Age, F(1, 23) = 3.22, *P* = .086, η^2^ₚ = .12, nor a significant interaction index of the OAB, F(1, 23) = 2.09, *P* = .16, η^2^ₚ < .08.

In line with Civile and McLaren (2022), we conducted an independent sample t-test comparing the FIE index (performance for upright minus inverted faces) for own-age/younger faces across the two tDCS conditions. This analysis showed that the FIE for younger faces in the anodal group was significantly reduced compared to the sham group, t(46) = 2.98, *P* = .004, η^2^ₚ = .16—like the effects observed for own-race faces in Civile and McLaren (2022).

Finally, we conducted a Bayes factor analysis based on Dienes (2011) to compare the OAB from the young adult sample in Civile and Wang (2025) with the eliminated OAB in our anodal tDCS group. This revealed a factor of 0.12, providing strong evidence for the null hypothesis (below the 0.30 cutoff; Jeffreys 1961, Jarosz and Wiley 2014), supporting the conclusion that anodal tDCS eliminates the OAB. We also conducted a further analysis on the FIE for own-age/younger faces in the sham vs anodal groups, using prior differences from Civile and McLaren (2022) that used Western Caucasian faces of a similar age range. This analysis yielded Bayes factor (BF) of 103, providing decisive evidence (greater than 100) that the tDCS reduces the FIE for own-age/younger faces (for further details, see the Supplemental Material Document).

## Discussion

The current study aimed to investigate the nature of the OAB by applying a tDCS procedure previously used to eliminate the ORB (Civile and McLaren 2022).

Results from the sham group confirmed the OAB, showing a larger FIE for own-age faces compared to other-age faces. In line with previous findings, this difference was primarily driven by lower recognition of upright other-age faces (Civile and Wang 2025). Importantly, in the anodal group, the FIE for own-age faces was significantly reduced compared to the sham group, eliminating the interaction index of the OAB. Further support comes from Bayesian factor analyses, which confirmed the reduction of the OAB in the anodal group by demonstrating a decrease in the FIE for own-age faces. We now have evidence that the same tDCS procedure, which affects the checkerboard inversion effect (Civile et al. 2016b) and the FIE (Civile et al. 2021a), can also influence the ORB (Civile and McLaren 2022) and the OAB. The common mechanism underlying these results is that the tDCS reduces the inversion effect for the most familiar stimuli, primarily driven by a decreased performance for upright stimuli.

In line with Civile and McLaren (2022)’s work on the ORB, our results could be explained by the MKM model of perceptual learning (MKM; McLaren et al. 1989, McLaren and Mackintosh 2000, McLaren et al. 2012). This model is based on the modulation of salience by error to produce the perceptual learning underlying the inversion effect for checkerboards (Civile et al. 2014b), and by extension, the FIE. It is parameterized such that if the features determining a stimulus are well predicted by other features (low error), then these features exhibit lower salience (activation). Conversely, features that are not well predicted, such as unique features, have higher salience. This approach facilitates discrimination between AX and BX when these stimuli are pre-exposed because the common features (X) would be better predicted than the unique features (A, B), leading to lower salience for the common features compared to the unique features.

We can extend this concept to prototype-defined stimuli, where the prototype can be considered as the set of common features, and the distinctive features of each exemplar are unique and thus differ among different exemplars. Familiarity with such a category leads to reduced generalization between exemplars because the influence of the common features present in each exemplar will diminish due to their reduced salience contingent on exposure to the category. This leads to perceptual learning since the salience of the unique features will be relatively high, resulting in improved discrimination. This process of feature salience modulation applies only to upright stimuli because participants have less experience with inverted stimuli; performance on these is not aided by significant perceptual learning.

We can apply this analysis to faces. Due to our experience with upright faces, the common facial features become strongly associated with one another, reducing their error scores, and consequently, the salience/activation of the units representing those features declines. In contrast, the novel/unpredicted features that are specific to a given face do not suffer from this reduction in salience. Thus, these ‘unique’ features stand out and become more available for learning, leading to improved discrimination among upright faces. However, when faces are inverted, this learning mechanism no longer applies because the unfamiliar spatial arrangement of features renders them less well predicted by other features, interfering with the salience modulation typically in place between common and unique features of upright faces.

When the anodal tDCS procedure is used, the reduction of the FIE would result from impaired performance for upright faces due to the stimulation disabling the salience modulation mechanism, which means that instead of prior pre-exposure to a prototype-defined category enhancing the discriminability of the exemplars from that category, it may now enhance generalization between them. Features common to exemplars become more prominent because they coactivate one another, while unique features have low salience as they do not receive additional activation. It is this change in perceptual learning that leads to a reduction in the inversion effect, as it diminishes participants’ ability to discriminate between upright faces, effectively making the faces appear more ‘similar’. Importantly, this explanation was supported by the simulations work featured in Civile et al. (2023) and Civile et al. (2025) and by the experimental work using the same tDCS procedure applied in a learning prototype-distortion task, where increased salience of the prototypical features within familiar stimuli led to improved task performance (McLaren et al. 2016).

One could argue that our results might also be explained by the social accounts. Based on the assumption that outgroup or ‘other’ faces (of different ages) are primarily processed in terms of prototypical features common across that outgroup, while ingroup (same-age) faces are processed in terms of their individuating information (Levin 2000, Hugenberg et al. 2013, Young et al. 2012), one could conclude that configural information is highly weighted for individuation. If prototypical features (e.g. the presence of wrinkles, skin tone, etc.) are not configural, then the disruption of configural processing due to inversion will be greater for ingroup faces than for outgroup faces. In turn, if tDCS impacts configural processing, it will mainly affect the processing of own-age faces. Moreover, even if similar expertise is present for both own-age and other-age faces, the social categorization account may suggest that this expertise is not fully utilized for other-age faces, as they are predominantly processed in terms of prototypical features rather than the individuating details that expert processing would support. Thus, if tDCS disrupts expertise-based processing, it would selectively impact own-age faces. Consequently, in both instances, we would expect tDCS to affect the OAB in a manner similar to our study.

Both explanations seem plausible; however, it is important to highlight that both assume a connection between the specific tDCS procedure and social categorization. So far, the literature has only systematically investigated the link between the tDCS procedure and the perceptual learning explanation, as demonstrated in the case of the inversion effect using non-social artificial stimuli that participants had no prior expertise with before entering the lab. Civile et al. (2014b) demonstrated that an inversion effect for prototype-defined checkerboards can be observed when participants are familiarized with them, as opposed to the lack of an inversion effect for checkerboards from a novel category or a familiar, non-prototype-defined category. Civile et al. (2016b), and Civile et al. (2021a), showed that the specific tDCS procedure reduces both the checkerboard inversion effect and the FIE in a similar manner. This has established a causal link between the two inversion effects. While social categorization explanations may offer an alternative framework (to be tested) for the effects observed here, it seems implausible for these to account for the tDCS effects on the checkerboards, especially given that these are completely non-social stimuli and the task is designed to control for the development of expertise. Future work should address this directly, for instance, by applying the same tDCS procedure to a study focused on a social-based inversion effect to determine whether similar effects are obtained.

It is important to note that our discussion of alternative social explanations assumes a general learning mechanism that accounts for the inversion effect in both newly acquired stimuli (e.g. checkerboards) and faces, where expertise develops over years of experience. While this provides a plausible interpretation for the current study, future research should examine how tDCS influences different levels of expertise based on familiarization (e.g. Gauthier and Tarr’s Greebles 1997) and distinct processes, such as image-based expertise versus face identity recognition (Bruce and Young 1986), as well as their relation to perceptual biases like ORB and OAB.

One final concern is that the reduced OAB in the anodal group could reflect mild adverse sensations causing distraction and lowering performance for younger faces. Future studies should address this directly, ideally using a within-subject tDCS design. However, consistent with previous work, anodal tDCS primarily affected upright younger faces compared to sham, aligning with the expertise account, while no significant effect was observed for inverted faces (see Supplemental Material). If distraction from anodal sensations were the cause, we would expect similar effects for inverted faces, especially since their performance was well above chance. Moreover, the same anodal procedure has previously enhanced performance in contexts where increased generalization benefits perceptual learning (e.g. Civile et al., 2020b; McLaren et al. 2016). If anodal sensations simply impaired performance, this pattern would appear across all perceptual learning tasks, which is not the case.

In conclusion, our results reveal that the tDCS protocol previously used to reduce the inversion effect for checkerboards and later found to reduce the inversion effect for own-race faces can similarly eliminate the OAB by affecting the recognition of own-age faces. This suggests a potential link between the effects of tDCS on perceptual expertise as a key mechanism underlying the OAB, as measured by the FIE.

More broadly, our findings contribute to the literature examining the effects of tDCS on face recognition, which varies in methodology across studies. For example, Pisoni et al. (2015) found that anodal tDCS targeting the T3 scalp area significantly reduced performance in a face–name association task compared to sham. Renzi et al. (2015) observed that although anodal tDCS did not modify the composite face effect, it exhibited a blocking effect on performance in tasks involving Mooney faces (black-and-white distorted faces), where participants often struggle with face detection (see also Yang et al. 2014). Conversely, Barbieri et al. (2016) found that anodal tDCS improved face and object recognition performance, although inversion was not tested.

Together, these studies, including our findings, highlight tDCS’s potential to modulate face recognition, ultimately advancing our understanding of the underlying cognitive mechanisms.

## Supplementary Material

nsag001_Supplementary_Data

## Data Availability

The data from the study are publicly available through the Open Science Framework (https://osf.io/bwf3s/). Stimuli and program code will be accessed by contacting the corresponding author (c.civile@exeter.ac.uk).
