# Supplementary material for: Transcranial direct current stimulation eliminates the own-age bias as indexed by the face inversion effect for own- versus other-age faces
Source: Soc Cogn Affect Neurosci. 2026 Jan 9;21(1):nsag001. doi: 10.1093/scan/nsag001 (PMC12884409; doi:10.1093/scan/nsag001)
Supplement: nsag001_Supplementary_Data [file nsag001_supplementary_data.zip › Supplemental Material Document.docx]

**Supplemental Material Document:**

**Transcranial Direct Current Stimulation (tDCS) eliminates the Own-Age Bias (OAB) as indexed by the Face Inversion Effect (FIE) for own- versus other-age faces.**

Ciro Civile and Guangtong Wang

Department of Psychology, Faculty of Health & Life Sciences,

University of Exeter, UK

**Materials**

The study utilized a set of high-resolution Western Caucasian faces from the FACES database (https://faces.mpdl.mpg.de/imeji). The set includes images of naturalistic faces of young and older women and men. The original stimuli were created by recruiting models with an “average” appearance, devoid of prominent features such as beards, tattoos, or piercings. The models included 60 young individuals (M = 24.3 years, SD = 3.5; age range 19–30), 60 middle-aged individuals (M = 49.0 years, SD = 3.9; age range 39–55), and 58 older individuals (M = 73.2 years, SD = 2.8; age range 69+), all of Western Caucasian background.

These models were trained to mimic six main facial expressions: neutrality, sadness, disgust, fear, anger, and happiness. The training involved phases for emotion induction (triggering spontaneous emotion) and controlled expression. High-quality digital photographs were then taken under consistent lighting conditions: 120° frontal lighting from above via a striplight for soft illumination, with additional 220° lighting from below, and white balance set to neutral gray. All images were color photographs with models directed to gaze directly at the camera.

Following stimulus development, only the most prototypical pictures of each model—rated by external coders—were selected for a validation study involving 154 participants (52 young, 51 middle-aged, and 51 older adults), who rated the faces on facial expression and perceived age. Based on this validation, the authors created a specific subset of faces representing younger, middle-aged, and older individuals (Ebner et al., 2010).

**D-Prime Calculations and Adjustments**

D-Prime (d’) sensitive measure indicates how well participants distinguish old from new stimuli across each stimulus type. A d' of 0 reflects performance at chance level. To calculate d’, we used participants’ hit rate (H)—the proportion of ‘YES’ responses to previously seen faces—and false alarm rate (F)—the proportion of ‘YES’ responses to new faces. Optimal performance maximizes H (reducing misses) and minimizes F (reducing false alarms); thus, the greater the difference between H and F, the higher the participant’s sensitivity. The measure d’ represents this difference and is calculated as the distance between the z-transforms of H and F: d’ = z(H) – z(F).

With d’ when someone achieves 100% accuracy, they correctly identify all signal and noise trials without errors. In this scenario, the d' value approaches infinity because it is calculated from the difference between the z-scores of hit rates and false alarm rates. Specifically, with perfect accuracy: Hit rate (H) = 1 and False alarm rate (F) = 0. Since the z-score of 1 is infinite and that of 0 is negative infinity, the d' becomes theoretically infinite, indicating perfect discriminability. Practically, due to mathematical limits, adjustments are made (e.g., corrections for perfect scores) to avoid infinite values. For H = 1, the adjustment is 1 - (1 / double the trials), and for F = 0, it is 1 / double the trials (Hautus et al., 2021).

In our study, 10 out of 384 H and F values were adjusted due to participants achieving 100% accuracy. Given the simplicity of the recognition task, it is unsurprising that participants scored perfectly on some trials.

**Analysis on Chance Performance**

We conducted a series of one-sample t-tests comparing the mean performance for each stimulus condition within each tDCS sample against the chance level, defined as d’ = 0 (equivalent to 50% accuracy) (see Table 1).

**Table.1.** Results of chance level performance for each stimulus condition.

**Additional Analysis between upright and inverted stimuli**

**To further examine the effects of tDCS, we conducted additional analyses comparing performance for upright and inverted faces across the two tDCS groups. A significant difference emerged for upright younger faces between the anodal and sham groups,**t**(46) = 2.14,**p**= .038, η²ₚ = .09. In contrast, no significant differences were found for inverted younger faces,** t**(46) = 1.42,**p**= .16, η²ₚ = .04, upright older faces,** t**(46) = 0.78,**p**= .43, η²ₚ = .01, or inverted older faces,** t**(46) = 0.65,**p**= .52, η²ₚ < .01.**

**Criterion Analysis**

A 2 × 2 × 2 mixed-model ANOVA with Orientation (upright vs. inverted) and Face Age (younger vs. older) as within-subjects factors, and tDCS Stimulation (sham vs. anodal) as a between-subjects factor revealed a significant main effect of Orientation, F(1, 46) = 25.57, p < .001, η²ₚ = .37, and of Face Age, F(1, 46) = 15.2, p < .001, η²ₚ = .25. No significant main effect of tDCS Stimulation was found, F(1, 46) = .39, p = .53, η²ₚ < .01. No significant interactions were observed for Orientation × tDCS Stimulation, F(1, 46) = 1.88, p = .17, η²ₚ = .03; Orientation × Face Age, F(1, 46) = .22, p = .64, η²ₚ < .01; or Face Age × tDCS, F(1, 46) = 0.94, p = .33, η²ₚ = .02, Orientation × Face Age × tDCS, F(1, 46) = .77, p = .78, η²ₚ < .01.


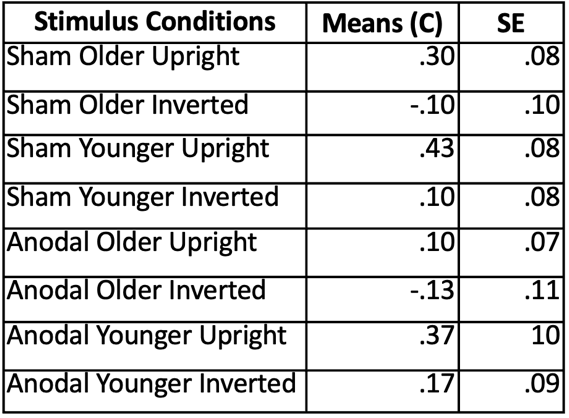


**Table.2.** Criterion means and SE for each stimulus condition.

**Bayes Factor Analyses**

Using the procedure outlined by Dienes (2011), we first conducted a Bayes analysis on the difference between the OAB found in previous work that adopted the same behavioral procedures and stimuli as our study, and the eliminated OAB in the anodal tDCS group in our research. We used the two-way interaction (Face Age x FIE) index of the OAB from the younger adult sample in Civile and Wang (2025) as the prior, setting the standard deviation of p (population value | theory) to the difference between the FIE score (upright – inverted) for own-age/younger faces versus the FIE score for other-age/older faces [0.48]. We used the standard error [0.14] and mean difference [-0.21] between the FIE score for own-age/younger faces and the FIE score for other-age/older faces in the anodal group from our study. We assumed a one-tailed distribution for our theory with a mean of 0. This resulted in a Bayes factor of 0.12, which provides strong evidence in support of the null hypothesis (less than 0.30 for the conventional cut-offs, see Jarosz & Wiley, 2014; Jeffreys, 1961), supporting the claim that the anodal stimulation procedure eliminates the OAB.

We conducted a further Bayes analysis on the FIE score for own-age/younger faces comparing the sham and anodal groups. We used as the *priors* the differences found in Civile and McLaren (2022), where the own-race faces were Western Caucasian and similar age range to the ones used in our study, setting the standard deviation of p (*population value | theory*) to the mean for the difference between the FIE for own-race faces in sham group vs that in the anodal group [0.36]. We used the *standard error* [0.18] and *mean difference* [0.62] between the FIE for own-age/younger faces in the sham group vs that in the anodal group. We assumed a one-tailed distribution for our theory and a mean of 0. This gave a Bayes factor of 103, which is decisive evidence (greater than 100) that these results demonstrate how the tDCS procedure used here reduces the FIE for own-age/younger faces.
